# Metformin, an Anti-diabetic Drug to Target Leukemia

**DOI:** 10.3389/fendo.2018.00446

**Published:** 2018-08-10

**Authors:** Giulia Biondani, Jean-François Peyron

**Affiliations:** Team #4: Leukemia: Molecular Addictions, Resistances & Leukemic Stem Cells, Université Nice Côte d'Azur, C3M-Inserm U1065, Paris, France

**Keywords:** metformin, leukemia, chemotherapy, adjuvant, AMPK pathway, metabolism and bioenergetics

## Abstract

Metformin, a widely used anti-diabetic molecule, has attracted a strong interest in the last 10 years as a possible new anti-cancer molecule. Metformin acts by interfering with mitochondrial respiration, leading to an activation of the AMPK tumor-suppressive pathway to promote catabolic-energy saving reactions and block anabolic ones that are associated with abnormal cell proliferation. Metformin also acts at the organism level. In type 2 diabetes patients, metformin reduces hyperglycemia and increases insulin sensitivity by enhancing insulin-stimulated glucose uptake in muscles, liver, and adipose tissue and by reducing glucose output by the liver. Lowering insulin and insulin-like growth factor 1 (IGF-1) levels that stimulate cancer growth could be important features of metformin's mode of action. Despite continuous progress in treatments with the use of targeted therapies and now immunotherapies, acute leukemias are still of very poor prognosis for relapse patients, demonstrating an important need for new treatments deriving from the identification of their pathological supportive mechanisms. In the last decade, it has been realized that if cancer cells modify and reprogram their metabolism to feed their intense biochemical needs associated with their runaway proliferation, they develop metabolic addictions that could represent attractive targets for new therapeutic strategies that intend to starve and kill cancer cells. This Mini Review explores the anti-leukemic potential of metformin and its mode of action on leukemia metabolism.

## Metformin: a tale of drug repositioning in cancer

Metformin is an active biguanide derivative extracted from the French Lilac (Galega officinalis), a plant discovered during the Middle Age for its healing effects on the diabetic condition. Metformin/Glucophage® was first prescribed in Europe in 1979, then in the United States by 1994 and is now the first-line treatment for type 2 diabetes (T2D) as more than 120 million patients are treated worldwide (1).

In 2001 metformin appeared on the cancer scene when it was observed that in hepatocytes it stimulated the AMP-activated serine threonine protein kinase (AMPK) (2), a sensor of the energetic cellular status and an important tumor suppressor pathway (3, 4).

This discovery prompted clinicians and researchers to measure cancer frequency in T2D patients under metformin. It was first shown in 2005 that metformin significantly reduced cancer incidence in a cohort of 983 T2D patients (5). Other studies confirmed that metformin was associated with a lower risk of cancer in treated diabetic patients (6–8).

These striking results led the renowned cancer researcher Lewis Cantley to consider that “Metformin may have saved more people from cancer deaths than any drug in history” (9).

Numerous investigations worldwide rapidly demonstrated direct anti-cancer effects of metformin on various models (10–12). *In vitro*, metformin exhibits a strong anti-proliferative action on cancer cell lines derived from breast, colon, ovaries, pancreas, lung, and prostate (13–15). These results were strengthened by pre-clinical *in vivo* experiments using xenografts or transgenic mice and chemically-induced cancers. As an example, in a tobacco-induced lung carcinogenesis mouse model, metformin decreases tumor burden by 72% (16). Evidences show that metformin can act through an AMPK dependent (17, 18) or independent (19) way. However, despite metformin is widely used in clinic, its molecular mechanism of action is still under debate.

## Metformin: mode(s) of action

From the different reports it appears that metformin exerts a double action at both organism and molecular levels.

### Metformin's systemic effects

Within the organism, metformin has an anti-hyperglycemic action but as it does not decrease insulin secretion there is no risk of hypoglycemia in normal subjects (20). In muscles, metformin reduces hyperglycemia through different mechanisms: by enhancing insulin-stimulated glucose uptake and reducing hepatic glucose output (21). It lowers the production of glucose by the liver, and increases glucose utilization by muscles and adipocytes. This results in a decreased insulinemia and an amelioration of insulin sensitivity, likely counteracting the increased glucose uptake by insulin, which facilitates tumor initiation and progression (22). It was thus envisioned that the anti-cancer effects of metformin could be due to its ability to reduce circulating levels of glucose and consequently of insulin and insulin-like growth factor 1(IGF-1) that are suspected to feed different cancers expressing the receptors for these growth factors on their surface (23–26).

Diabetes, in particular T2D, and obesity are clearly associated with an increased risk to develop various cancers (27). However, no increased incidence was observed for hematologic malignancies (28) suggesting at first that the systemic effects of metformin may not apply to leukemia. Nevertheless, a metabolic syndrome with insulin resistance has been reported in leukemic patients exposed to high dose glucocorticoids (29). This could favor a therapy-induced obesity with hyperinsulinemia that supports leukemic cell survival and worsens patient's outcome. Insulin and IGF-1 receptors were found expressed on acute lymphoblastic leukemia (ALL) and acute myeloid leukemia (AML) (30, 31) and insulin stimulates *in vitro* the proliferation of ALL cell lines and primary cells that were sensitive to metformin (32). At the molecular level, an IGF1-IGF-1R autocrine loop is responsible for activation of a leukemia-supportive PI3K/Akt/mTOR pathway (33). Pharmacological interference with the insulin receptor and/or IGF1R autocrine loops affects leukemic proliferation (34) and potentiates the apoptotic action of etoposide (31). Similarly, targeting IGF-1R interferes with the growth of chronic lymphocytic leukemia (CLL) (35).

If insulin/IGF-1 do not appear to be strong oncogenic drivers for acute leukemias, they are likely trophic factors, supporting the rational use of metformin to decrease hyperinsulinemia and to indirectly affect leukemic cells.

### Metformin's molecular effects

**Figure 1:**
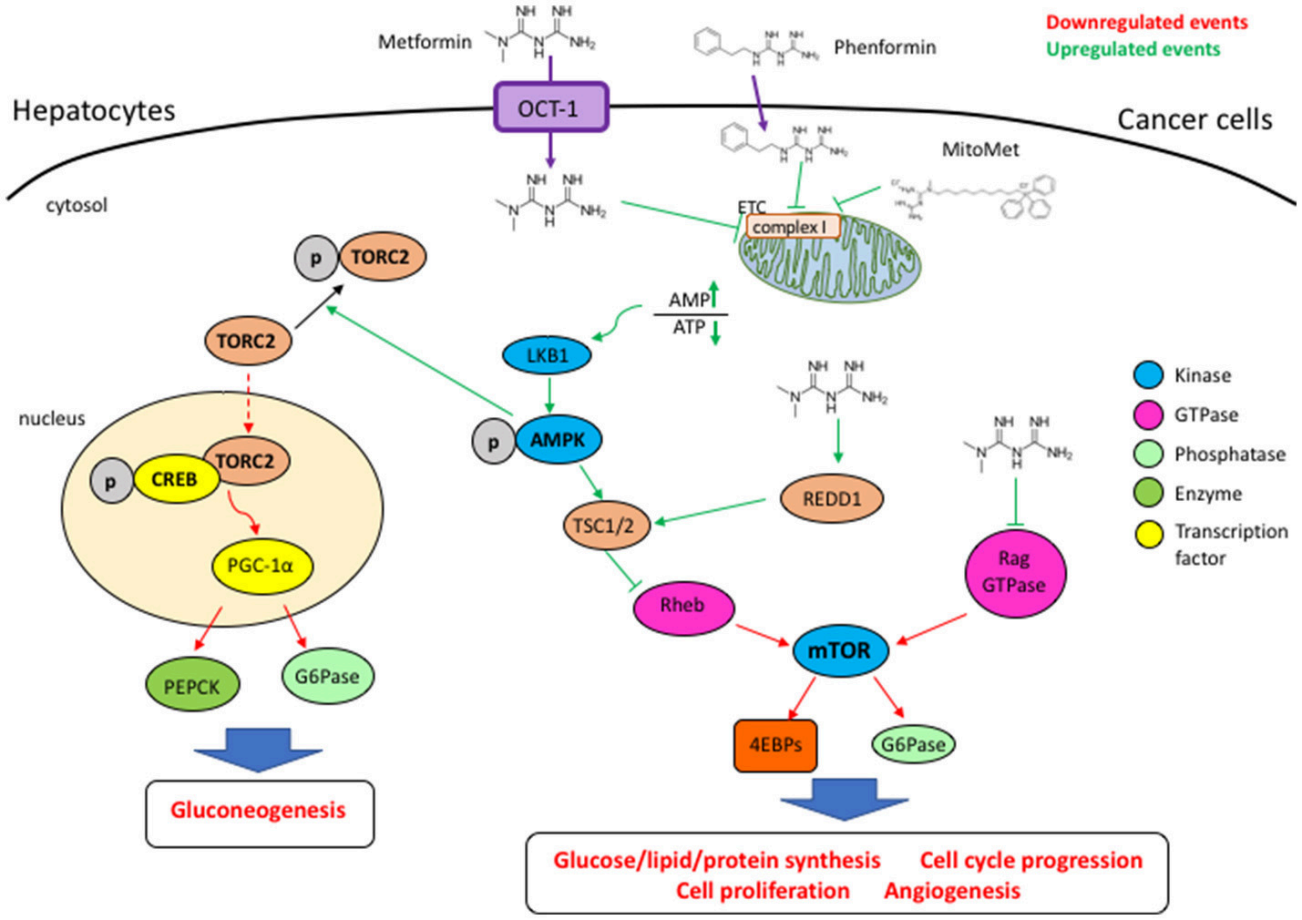
The entrance of metformin is mediated by the OCT1 transporter. By blocking the mitochondrial respiratory chain complex I, metformin and phenformin determine an increase of the AMP/ATP ratio, a condition that activates AMPK through phosphorylation by LKB1. Metformin-activated AMPK counteracts the activation of the mTORC1 complex, impairing cell cycle progression and proliferation, angiogenesis, as well as lipid and protein syntheses. Metformin can induce REDD1 and inhibit Rag GTPases, thus leading to the blocking of mTORC1 through an AMPK-independent way. In an AMPK-dependent way metformin promotes TORC2 phosphorylation and blocks its nuclear translocation, its association with phospho-CREB, impairing the transcription of genes such as PGC-1α, G6Pase, and PEPCK whose products promotes gluconeogenesis.

As shown in Figure 1, metformine inhibits oxidative respiration by acting on the complex I of the mitochondrial respiratory chain (17, 18), leading to a drop in ATP synthesis, tilting the AMP/ATP balance toward AMP, with the consequent stimulation of AMPK. It is well known that the LKB1/AMPK pathway also regulates the protein synthesis rate through the control of mTOR. Activated AMPK stimulates tuberous sclerosis complex 1/2 (TSC1/2) through phosphorylation and its GTPase-activating protein (GAP) function toward the small G-protein Rheb (Ras homolog enriched in brain), thus determining the switching off of Rheb and resulting in the inhibition of mTOR activity (36–38). AMPK activation requires binding and phosphorylation by the tumor suppressor liver kinase B1 (LKB1) (39, 40). Therefore, the absence of LKB1 impedes an AMPK-negative regulation of cancerous cell metabolism.

The AMPK pathway is a major repressor of the mTOR pathway that uses energy and nutrients to stimulate ATP-consuming anabolic reactions, favoring growth and proliferation (41). Activation of the PI3K/Akt pathway, a major upstream activator of mTOR, is restrained by the lipid phosphatase and tumor suppressor PTEN (phosphatase and tensin homolog), frequently inactivated in cancer (42, 43). Defects in control by PTEN lead to a constitutive activation of the Akt pathway that is involved in the etiology of various pathological conditions such as diabetes, aging, and cancer (44).

The mTOR serine/threonine kinase is the active central component of the mTORC1 and mTORC2 cellular complexes that function to coordinately stimulate cell growth (44). mTORC1 is crucial for the synthesis of proteins, lipids, and nucleic acids while mTORC2 phosphorylates Akt to stimulate proliferation and survival (45, 46). Furthermore, AMPK promotes phosphorylation of TORC2 (transducer of regulated CREB activity 2) to block its nuclear translocation and association with phospho-CREB (CRE binding protein), thus impairing the transcription of genes involved in gluconeogenesis such as peroxisome-proliferator-activated receptor-γ co-activator-1α (PGC-1α), glucose-6-phosphatase (G6Pase), and phosphoenolpyruvate carboxykinase (PEPCK) (38, 47).

Through AMPK stimulation, metformin interferes with mTORC1 activation. In addition, AMPK inhibits ATP formation through fatty acid oxidation (FAO) (48) and stimulates glycolysis by phosphorylation-induced activation of phosphofructo-2-kinase (PFK2) (49). Also, AMPK modulates gene expression for important metabolic enzymes (50) and induces a metabolic cell cycle checkpoint through p53 activation (51). Therefore, AMPK agonists as well as indirect activators such as metformin can be envisioned as promising anti-cancer compounds.

### AMPK-independent effects of metformin

Not all actions of metformin are mediated by AMPK (19). Metformin together with hexokinase 2 (HK2) depletion synergistically interferes with mTORC1 activation through the induction of the mTORC1 inhibitor REDD1 (regulated in development and DNA damage) in hepatocellular carcinoma cells, even upon depletion of AMPKα1 and AMPKα2 (52). Repression of G6Pase and of hepatic glucose production by metformin still occurs in both AMPK and LKB1-deficient hepatocytes (53).

In AML cells metformin can block proliferation at either G0/G1 or S-G2/M, depending on the cell line analyzed. Furthermore, by using a siRNA of AMPKα1/2, Scotland and colleagues showed that metformin-induced cell death is not dependent by AMPK activation in AML cells (54). In prostate cancer cell lines, metformin has AMPK-independent anti-proliferative effects through induction of REDD1 (55). In breast cancer cells metformin interferes with purine/pyrimidine and glutathione synthesis upstream of AMPK (56).

Metformin and resveratrol synergistically block pancreatic cancer cell proliferation *in vitro* and *in vivo* by inhibiting vascular endothelial growth factor B (VEGF-B) signaling pathway (57).

## Leukemia

Leukemia represent 2.8% of all cancers and 3.4% of deaths from cancer worldwide, with 351,000 new cases/year. Leukemia results from the transformation of hematopoietic stem-progenitor cells (HSPCs). Acute lymphoid or myeloid leukemia (ALL/AML) show an intense proliferation of immature leukemic blasts arrested at various stages of differentiation (58, 59). Despite important progress in treatments, the 5-year survival for T-ALL is 70–75% for children and only 35–40% for adults (59). New therapeutic strategies should therefore be identified to eradicate leukemia.

### Finding new therapeutic options for leukemia

Targeting the energetic metabolism of cancer cells is emerging as an attractive option (60) as cancer/leukemic cells reprogram their metabolism to fulfill their intense metabolic needs. Consequently, they develop metabolic addictions that can be used as new targeting options to starve and kill them (60).

### The PI3K/Akt/mTOR axis supports leukemic growth

The control of the PI3K/Akt/mTOR axis by PTEN is fundamental for the self-renewal of HSCs and PTEN knock-out generates leukemia in mice (61). A common biochemical feature among acute leukemia is the abnormal and constitutive activation of the PI3K/Akt/mTOR pathway (62, 63). Separated or combined pharmacological targeting of PI3K, Akt, or mTOR triggers leukemic cell death in AML and ALL (64, 65). PI-103, a dual inhibitor of PI3K and mTOR displays anti-leukemic properties (66). Unfortunately, the immunosuppressant rapalogs (Temsirolimus, Everolimus) that target mTORC1 activation, showed a limited anti-cancer activity as they failed to inhibit mTORC2 activity and reactivated the tumor supportive Akt pathway (41, 67, 68). Torkinibs, ATP-competitive inhibitors of the mTOR kinase activity, target both mTOR complexes (69, 70) and have already displayed promising anti-cancer properties on leukemia models (71–73).

### Metformin: a new treatment for leukemia?

Metformin represents an interesting opportunity to target leukemia through inhibition of constitutive mTOR, a pathological hallmark in leukemogenesis. In 2010 metformin was shown to interfere with AML proliferation and clonogenic activity and to induce apoptosis in human immortalized cell lines and primary samples while it did not affect normal CD34+ HSCs (37). Metformin, after blocking mTORC1 activation, prevents initiation of translation, in particular of c-myc, cyclin D1, and Bcl-xL that are crucial for cancer proliferation (37). Metformin induces apoptosis of leukemic megakaryoblasts from acute megakaryoblastic leukemia (AMKL) which is a rare type of leukemia with poor prognosis (74). Metformin could be an option for the DNA repair defective Fanconi Anemia pre-leukemic disorder as it is toxic after inhibiting the respiratory chain (75).

In T-ALL cells, metformin stimulates AMPK to inhibit mTOR and trigger an autophagic response that precedes apoptosis. By affecting protein synthesis, metformin strongly decreases c-myc and Bcl-xL levels (76). This apoptotic action of metformin in T-ALL also involves an AMPK-dependent activation of the ER stress/unfolded protein response (UPR) (77). In this model, metformin induces a compensatory, anti-apoptotic activation of Akt and of PIM-2, that could be reversed by inhibitors, synergizing with metformin for cell death induction.

Genetic defects in the PTEN tumor suppressor gene are leading to the constitutive activation of the PI3K/Akt/mTOR pathway in T-ALL (78) and are associated with a poor outcome in pediatric T-ALL (79). Tumor cells from a mouse T-ALL model generated by the T-cell specific deletion of PTEN display a constitutive activation of PI3K/Akt/mTOR that could be inhibited by metformin through AMPK activation and by torkinibs (80). Deletion of LKB1 in mice with a PTEN+/- background increases lymphoma incidence that appeared with a shorter latency and were sensitive to metformin (81).

Metformin counteracted the activation of the PI3K/Akt/mTOR pathway triggered by several oncogenes such as the Bcr-abl fusion tyrosine kinase in CML and Phi+ T-ALL and B-ALL and the Tax oncoprotein in HTLV-1-induced ATL (human T-lymphotropic virus type 1-induced adult-T-cell leukemia). Through AMPK activation, metformin suppresses proliferation and clonogenic activity of various CML lines, including those expressing the imatinib-resistant T315I Bcr-abl mutant (82). In ATL, LKB1/AMPK activation by metformin inhibits leukemic proliferation by reducing Tax expression (83). In CLL, metformin prevents cell cycle entry of leukemic cells *in vitro* after engagement of a CD40-CD40L proliferative stimulus (84). CLL cells that are sensitive to the tyrosine kinase inhibitor dasatinib appears to be selectively killed by metformin (85).

Leukemic stem cells (LSCs) are the rare cells at the origin of leukemia and also of relapse because of their intrinsic mechanisms of resistance to chemotherapies (86). Interestingly, in T-ALL metformin targets the Hoescht 33342^low^ side population and the CD34+CD7-CD4- subset that are known to be enriched in LSCs (76). Similarly, cancer stem cells (CSCs) in different solid tumors appears to be highly sensitive to low doses of metformin (87, 88).

### Metformin in combination therapies

The eradication of cancer will require the combination of multiple therapeutic strategies in a personalized manner. Anti-cancer clinical protocols and drug cocktails would need to be adapted to the specific genetic defects of each patient. Nevertheless, targeting a common dysregulated cellular function such as the reprogrammed cancer metabolism with a metabolic disruptor such as metformin is likely to be an interesting adjuvant approach.

Metformin has already been associated to several classical chemotherapeutic drugs with promising results. Metformin shows additive effects with anthracyclines (doxorubicin, daunorubicin) to reduce growth and survival of lymphoma cells (80), T-ALL cells (89), and ALL (32). The use of metformin could help to reduce the dose of doxorubicin necessary to prolong remission (88) and consequently to reduce cardiac toxicity of anthracyclines.

In T-ALLs metformin synergizes with dexamethasone, the glucocorticoid used as first line treatment for acute leukemias (80), and also potentiates the effect of the microtubule-disrupting agent vincristine (90) and of the topoisomerase II inhibitor etoposide (32).

All-trans retinoic acid (ATRA) is used in acute promyeloid leukemia (APL) to overcome the differentiation block induced by the PML-RAR fusion oncoprotein. By inducing PML-RAR degradation, metformin synergized with ATRA to induce APL cell death (91).

Triggering leukemia apoptosis at the mitochondrial level with the bcl-2 inhibitor ABT-737 is a promising therapy which was shown to be enhanced by metformin-induced mitochondrial membrane depolarization (92). The anti-leukemic activity of the Flt3 inhibitor sorafenib, that was developed to target poor prognosis-ITD Flt3 AML cells, could be enhanced by metformin, thus inducing a strong decrease in the expression of several components of the mTOR pathway (93).

### Metformin and other metabolic disruptors

In several studies metformin displays strong potentiating effects when combined with molecules affecting metabolism, in particular glycolysis, such as ritonavir in multiple myeloma (MM) (94) and CLL (95). *In silico*, metformin was predicted to combine with an inhibitor of the Glut4 glucose transporter to affect MM (96).

Disruption of the mitochondrial respiratory complex I by metformin is followed by a compensatory upregulation of glucose uptake and glycolysis (97, 98). As a consequence metformin was shown to synergize with the non-metabolizable glucose analog and hexokinase inhibitor 2-deoxy-glucose (2-DG) in T-ALL (80) and in CML (99) and with the glycolysis inhibitor sodium dichloroacetate (DCA) in B-CLL (100). Similar effects have been observed in Flt3-positive AML when metformin was associated with the metabolic inhibitor 6-BT (101). Cell death induction of MM cells after disrupting protein homeostasis with the proteasome inhibitor bortezomib can be enhanced by metformin, preventing a protective autophagic response (102). ALL cells display a metabolic dependency on asparagine that can be targeted with L-asparaginase, an effect further amplified by metformin (80).

### What is better: targeting the warburg's effect or mitochondria in leukemia?

In the 1920s, Otto Warburg and colleagues observed for the first time that cancer tissues were taking up enormous amounts of glucose compared to the surrounding tissue. Later, in 1956 Otto Warburg proposed that cancer cells have defective mitochondria because they utilize glucose through aerobic glycolysis, unlike normal cells which use glucose to produce ATP through oxidative phosphorylation (OXPHOS) in mitochondria (103). It was realized a couple of years ago that despite a far less efficient ATP production, this metabolic reprogramming represents an adaptation to optimize the utilization of nutrients to produce the biomass necessary for the generation of new proliferating cancer cells (104–106). Nevertheless, cancer and leukemic cells need active mitochondria for their fitness. Targeting the mitochondria respiratory function by inhibiting the electron transport chain (80), mitochondrial translation (107), or the FAO (108), are all new efficient approaches to kill leukemic cells. Recently, a mitochondrial transfer from stromal cells toward leukemic AML cells provided them with a survival advantage toward chemotherapy (109, 110). An important metabolic plasticity appears to take place as the environment of leukemic cells is changing (111). AML cells can become more sensitive to metformin when cultured in low-glucose medium or after downregulating glycolysis with 2-DG or an Akt inhibitor (54). Similarly, pharmacological approaches to inhibit OXPHOS markedly enhanced the anti-leukemic effects of cytarabine (112).

### Metformin for cancer patients: dose and effects

There are at least two important questions pending about the use of metformin in cancer.

First, will the ability of metformin to control hyperinsulinemia and glycemia in T2D patients stand for non-diabetic people? The 306 registered clinical trials on metformin and cancer will provide important answers. In relation to this review, metformin is tested (NCT01324180) in relapsed childhood ALL in association with vincristine, dexamethasone, doxorubicin, and PEG-asparaginase that are classical drugs for these leukemias. Metformin will be evaluated as a monotherapy for untreated or relapsed CLL patients in a phase 2 pilot study (NCT01750567).

Second, the doses of metformin that are efficient *in vitro* on cancer models are in the mM range, far above those obtained in treated T2D patients (6–30 μM) (113, 114). The cellular entry of the highly hydrophilic metformin is limited by expression of the organic cation transporter (OCT) (115). Interesting areas of research aim at facilitating metformin uptake through specific encapsulation, use of nanocarriers, or after chemical modifications. Coupling a mitochondrial vector to metformin (MitoMet) increases its ability to interfere with OXPHOS and consequently its efficiency to affect proliferation and to trigger ROS-dependent apoptosis in pancreatic cancer *in vitro* and *in vivo*, without affecting normal fibroblasts (116).

Phenformin, a hydrophobic metformin derivative is more active than metformin (81, 117) but was rapidly withdrawn from the market in the late 1970s because of numerous deadly cases of lactic acidosis. We now believe that phenformin could be worth testing as an adjuvant molecule for cancer patients with a monitoring of lactic acidosis. A clinical trial (NCT03026517) will evaluate phenformin in combination with dabrafenib and trametinib for patients with BRAF-mutated melanoma.

Recently Higurashi et al demonstrated the important role of metformin in chemoprevention of colorectal cancer (118). Other clinical trials are ongoing for coloncancer and other tumor types (e.g., NCT03047837; NCT02581137; NCT01312467; NCT01579812; NCT02581137).

## Conclusions and perspectives

Many studies support to use metformin and derivatives like phenformin as global adjuvants for classical anti-leukemic drugs. Improving metformin entry and access to its cellular target(s) through chemical modifications or the use of nanocarriers could be important means to increase the potential of this interesting anti-metabolic molecule.

## Author contributions

GB and J-FP equally wrote the article. GB did the illustration work.

### Conflict of interest statement

The authors declare that the research was conducted in the absence of any commercial or financial relationships that could be construed as a potential conflict of interest. The handling editor declared a shared affiliation, though no other collaboration, with the authors at time of review.
